# Association between *Helicobacter pylori* infection and colorectal polyps

**DOI:** 10.1097/MD.0000000000035591

**Published:** 2023-10-20

**Authors:** Nergis Basmaci, Ali Karataş, Mustafa Ergin, Giyaseddin Şükrü Dumlu

**Affiliations:** a Afyonkarahisar Dinar State Hospital, Department of Internal Medicine, Afyonkarahisar, Turkey; b Gazi University Faculty of Medicine, Department of Gastroenterology, Ankara, Turkey; c Aksaray Training and Research Hospital, Department of Gastroenterology, Aksaray, Turkey.

**Keywords:** adenoma, colonoscopy, colorectal polyp, esophagogastroduodenoscopy, *Helicobacter pylori*

## Abstract

It was aimed to investigate whether the *Helicobacter pylori* infection is related to the frequency, localization, size and number of colorectal polyps. The data of 4561 patients who underwent esophagogastroduodenoscopy and colonoscopy were analyzed retrospectively. Patients with and without polyps at colonoscopy were grouped and the frequency of *H pylori* infection was compared in these patients. The relationship between the groups was evaluated with statistical methods. It was determined that the rate of *H pylori* infection was higher in patients with colorectal polyps than in patients without polyps (*P* < .005). Patients with multiple polyps, polyps larger than 1 cm, and tubulovillous and villous adenoma from polyp types had a higher rate of *H pylori* infection (*P* = .095; *P* .004; *P* .001). When the polyps were evaluated according to their localization, *H pylori* infection rates were not different between the groups (*P* = .341). It has been observed that the rate of *H pylori* infection is higher in large polyps, multiple polyps, tubulovillous and villous adenomas, which are known to have a higher risk of malignancy.

## 1. Introduction

Colorectal cancer (CRC) is one of the most common causes of death worldwide.^[1]^ Its morbidity tends to increase in developed and developing countries.^[2]^ According to 2020 GLOBOCAN cancer and death rates, 19.3 million new cancer cases were reported worldwide in 2020 and approximately 10 million deaths occurred due to these cancers. The global cancer burden is expected to be 28.4 million cases in 2040. Colorectal cancer is the third most common cancer (10%) after breast and lung cancer, and it ranks second after lung cancer in terms of mortality.^[3]^ According to our country’s data, colorectal cancer ranks third in both women and men, similar to global data. Its frequency is 24.8 per hundred thousand in men and 14.7 per hundred thousand in women. Approximately 18,750 people are diagnosed with colorectal cancer in a year in Türkiye.^[4]^ Colorectal adenomatous lesions are defined as precancerous lesions. The frequency of colorectal polyps in Türkiye was found to be 31%. Eighty percentage of these are adenomatous polyps.^[5]^ Early diagnosis and treatment of colorectal adenomatous polyps significantly reduce the incidence of CRC.^[6,7]^

*Helicobacter pylori* infection is one of the most common bacterial infections in the world.^[8]^ More than 50% of the world’s population and 70% to 80% of the population in developing countries are thought to be infected with *H pylori*.^[8,9]^

In some studies, it has been observed that there is a relationship between *H pylori* infection and colonic neoplasms. Meta-analyses show that *H pylori* infection increases the risk of colorectal adenoma by 1.4 to 1.6 times.^[10]^ Another large-scale study showed the relationship between *H pylori*-positive gastritis and colonic neoplasms.^[11]^ Another study reported from Korea revealed that *H pylori* infection is an independent risk factor for colonic adenomatous polyps.^[10]^

Individuals infected with *H pylori* have chronic gastric inflammation and an immunoinflammatory response to the host’s gastric cells. Due to this response, gastrin secretion increases. Because of hypergastrinemia, acid secretion increases and, accordingly, hyperproliferation develops in the epithelial cells of the gastrointestinal tract. Gastrin levels have also been shown to be high in patients with colorectal cancer.^[12]^

In addition, *H pylori* can change the type and content of intestinal flora and increase ammonia levels. It is known that high luminal ammonia levels are associated with colorectal cancer.^[12]^

In addition to this information, it seems that there are not enough studies on the relationship between *H pylori* infection and colorectal polyp subtypes. This study was planned because the incidence of CRC is on an increasing trend in our country as well as in the rest of the world, and the prevalence of *H pylori* is still high in our country. In this study, it was aimed to investigate whether the frequency of *H pylori* infection is related to the frequency, localization, size and number of polyps.

## 2. Material and method

### 2.1. Study design and patient selection

Patients who underwent esophagogastroduodenoscopy and colonoscopy between March 2015 and March 2019 in Gazi University Faculty of Medicine Gastroenterology Department Endoscopy Unit were included in the study. Ethics committee approval was obtained from Gazi University Scientific Research Ethics Committee before starting the study. Esophagogastroduodenoscopy and colonoscopy were performed on the specified dates, biopsies were examined by performing polypectomy during colonoscopy, the presence of *H pylori* was evaluated by endoscopic biopsy and stool evaluation for *H pylori* antigen, and patients over 18 years of age were included in the study. Rapid urease test was performed on gastric biopsy samples for histological examination.

Patients who are not suitable for gastric emptying and bowel cleansing before esophagogastroduodenoscopy and colonoscopy procedures, patients who cannot tolerate the procedure, patients diagnosed with gastrointestinal (GI) tract carcinoma by biopsy or who were followed up for GI system carcinoma before, patients undergoing endoscopy due to GI bleeding, patients whose proton pump inhibitor (PPI) therapy was not discontinued 2 weeks before the procedure, or patients who had received *H pylori* eradication therapy or antibiotic therapy for other reasons < 4 weeks ago were excluded from the study.

In patients where polyps were observed during colonoscopy, polypectomy was performed using hot or cold snare polypectomy or forceps, depending on the location, and the polyps were completely removed and examined histologically. Polyp sizes were measured by comparing them with the size of the forceps during the procedure, and the length of the pathological specimens after polypectomy was measured with a surgical ruler and recorded.

**Figure 1.**
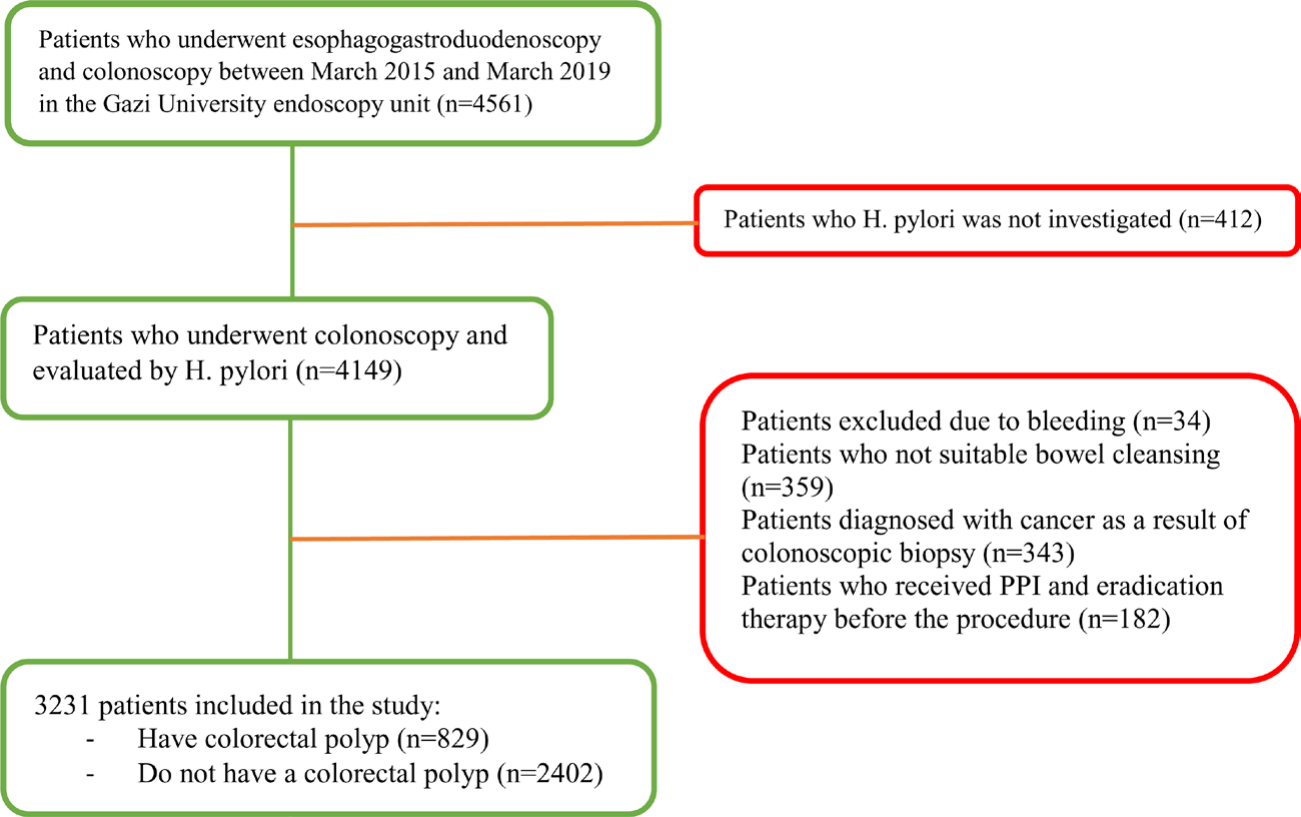
Study design.

The records of 4561 patients who underwent esophagogastroduodenoscopy and colonoscopy on the specified dates were scanned from the electronic archive. In 359 of these patients, bowel cleansing was not appropriate or the patient could not tolerate the procedure. Three hundred forty-three patients were excluded from the study because of invasive carcinoma in the polyp biopsy result at colonoscopy. Thirty-four patients who underwent endoscopy due to GI bleeding were excluded from the study. One hundred eighty-two patients were excluded from the study because they received PPI treatment 2 weeks before or *H pylori* eradication treatment 4 weeks before the procedure. Although polyps were seen on colonoscopy in 412 of the patients, the presence of *H pylori* was not investigated in these patients. Of the 3231 patients included in the study, 829 had colorectal polyps, and 2402 did not have polyps (Fig. 1).

### 2.2. Pathological evaluation

Esophagogastroduodenoscopy and colonoscopy were performed at the same or different times in all 3231 patients included in the study. Polypectomy was performed on patients with polyps in the colon, and the samples were evaluated by the pathology department. Polyps are pathologically classified as tubular, tubulovillous, villous adenoma, hyperplastic polyp, sessile serrated polyp, and inflammatory polyp. Polyps are grouped according to their size as being smaller than 0.5 cm, between 0.5 to 1 cm and larger than 1 cm. The presence of *H pylori* was determined by endoscopic biopsy samples or stool serology.

### 2.3. Statistical analysis

Patients with and without polyps at colonoscopy were grouped and the frequency of *H pylori* infection was compared in these patients. For numerical data, the Student *t* test and Mann–Whitney *U* test were used, and the chi-square test was used for categorical data comparisons between groups. Multivariate analysis was performed in patients with polyps and *H pylori* infection, and the relationship between colonic polyp and *H pylori* was evaluated in terms of polyp type, number and size. Statistical significance was defined as *P* < .05. IBM SPSS version (SPSS 15.00) program was used while evaluating the study data.

## 3. Results

### 3.1. Demographic features

53.5% of the patients (n = 1727) were female; 46.5% (n = 1504) were male. Colorectal polyps were detected in 829 (25.7%) of 3231 patients included in the study. Polyps were not detected in 2402 patients (74.3%).

The mean age of patients with polyps was 60.57 ± 10.23, and the mean age of patients without polyps was 54.96 ± 14.68 years. When the mean ages of patients with and without polyps were compared with the *t* test, the difference between them was found to be statistically significant. The demographic characteristics of the patients included in the study are shown in Table 1.

**Table 1:** Demographic characteristics of the patients included in the study.

	Number (n)	(%)
Gender (n = 3231)		
Female	1727	53,5
Male	1504	46,5
Excistance of polyp (n = 3231)		
Having a polyp	829	25,7
No polyp	2402	74,3
In patients with polyps (n = 829)		
Female	351	42,3
Male	478	57,7
In patients with polyps (n = 829)		
Single polyp	335	40,4
Multiple polyps	494	59,6
According to the polyp size (n = 829)		
Smaller than 0.5 cm	540	65,1
Between 0.5 cm–1 cm	185	22,3
Larger than 1 cm	104	12,6
According to the histopathological types (n = 829)		
Tubular adenoma	433	52,2
Tubulovillous adenoma	114	13,8
Villous adenoma	50	6
Hyperplastic polyp	198	23,9
Hyperplastic polyp + adenoma	12	1,4
Sesile serrated polyp	14	1,7
Inflammatory polyp	8	1
According to the localization (n = 829)		
Ascending colon	115	13,9
Transverse colon	123	14,8
Descending colon	146	17,6
Sigmoid colon	223	26,9
Rectum	178	21,5
Rectosigmoidal junction	44	5,3
Existance of *H pylori* infection (n = 3231)		
*H pylori* infection exist	1105	34,2
*H pylori* infection does not exist	2126	65,8
In patient with *H pylori* infection (n = 1105)		
Female	597	54
Male	508	46

### 3.2. *H pylori* infection rate

The rate of *H pylori* infection in patients with polyps was higher than in patients without polyps (45.6% vs 30.3%; *P* < .001). *H pylori* infection was detected in 48% (n = 237) of patients with multiple polyps and 42% (n = 146) of patients with single polyps (*P* = .095). In the logistic regression analysis, it was observed that *H pylori* infection was 1.6 times more common in patients with a single polyp, and 2.1 times more common in patients with multiple polyps compared to those without polyps (Table 2).

**Table 2:** Relationship between *H pylori* infection and colorectal polyp.

	*H pylori* negative		*H pylori*-positive		*P* value*	OR (CI %95)
	Number	%	Number	%		
Existance of polyp (n = 3231)						
Having a polyp	1675	69.7	727	30.3	<.001 (X² = 64.367)	
No polyp	451	54.4	378	45.6		
Number of polyp (n = 829)						
Single polyp	194	58	237	42	= .095 (X² = 2788)	1.67 (1.32–2.11)
Multiple polyp	257	52	1105	34.2		2.12 (1.74–2.58)

CI = confidence interval, OR = odds ratio.

* Chi-square test.

When the different polyp types were examined separately, it was observed that there were significant differences in the frequency of *H pylori* infection. When tubulovillous and villous adenomas are compared with other polyp types (tubular, hyperplastic, sessile serrated polyp, inflammatory polyps); *H pylori* infection rate is found to be higher in tubulovillous and villous adenomas compared to other polyp types, and this difference is statistically significant (58.5% vs 42.4%; *P* < .001). According to the logistic regression analysis, it was observed that the rate of *H pylori* infection was 1.9 times higher in tubulovillous and villous adenomas compared to other polyp types (odds ratio = 1.917; 95% confidence interval 1.356-2.712). When tubulovillous adenoma and other polyp types were compared, it was observed that the frequency of *H pylori* infection was higher in patients with tubulovillous adenoma than in other polyp types (*P* = .016; X²=13.887). When other polyp types were compared among themselves, no significant difference was found in terms of the frequency of *H pylori* infection (*P* = .163; X²=6.521). Comparisons made according to polyp types are summarized in Table 3.

**Table 3:** Comparison of polyp types and *H pylori* rates.

	*H pylori* negative		*H pylori*-positive		*P* value*	OR (CI %95)
	Number	%	Number	%		
Comparison of tubulovillous + villous adenoma and other polyp types (n = 829)						
Tubulovillous and villous adenoma	68	41.5	96	58.5	< .001 (*X*²=13.799)	1.91 (1.35–2.71)
Other polyp types	383	57.6	282	42.4		
Comparison of other polyp types among themselves (n = 665)						
Tubular adenoma	236	54.5	197	45.5	.163 (*X*² = 6521)	
Hyperplasric polyp	124	62.6	74	37.4		
Sesile serrated polyp	10	71.4	4	28,6		
Inflamatory polyp	4	50	4	50		
Hyperplastic polyp + adenoma	9	75	3	25		
Comparison of tubulovillous adenoma and other polyp types (n = 779)						
Tubulovillous adenoma	50	43.9	64	56.1	.016 (*X*² = 13.887)	
Other polyp types	383	57.6	282	42.4		

CI = confidence interval, OR = odds ratio.

* Chi-square test.

When the polyps were evaluated according to their size, it was seen that 43.7% of the patients with polyps smaller than 1 cm and 58.7% of the patients with polyps larger than 1 cm had *H pylori* infection, and this difference was statistically significant (%43.7 vs %58.7; *P* < .004; X²=8173). Polyps larger than 1 cm had a 1.82-fold increase in *H pylori* infection compared to polyps smaller than 1 cm (odds ratio 1.826; 95% confidence interval 1.203–2.770) (Table 4). When the polyps were evaluated according to their localization, no statistically significant relationship was found between the groups in terms of the frequency of *H pylori* infection (*P* = .341; X²=5.654).

**Table 4:** The relationship between polyp size and *H pylori.*

	*H pylori* negative		*H pylori*-positive		*P* value*	OR (CI %95)
	Number	%	Number	%		
According to polyp size (n = 829)						
<1cm (n = 725)	408	56.3	317	43.7	.004 (*X*² = 8173)	1.82 (1.2–2.7)
>1cm (n = 104)	43	41.3	61	58.7		

CI = confidence interval, OR = odds ratio.

* Chi-square test.

## 4. Discussion

In our retrospective, single-center study, a significant relationship was found between gastric *H pylori* infection and the frequency of colorectal polyps. According to the results obtained from the study; *H pylori* infection rate was found to be higher in patients with polyps compared to patients without polyps; this difference was observed to be more pronounced in polyps larger than 1 cm, tubulovillous adenoma and villous adenoma. Although the study was single-centered, a reasonable number of cases were reached. The relationship between the frequency of *H pylori* infection and the type, number, size and localization of polyps was examined in detail. The association of *H pylori* infection with both adenomatous and non-adenomatous polyps was investigated. Patients who used antisecretory drugs and antibiotics prior to the presence of *H pylori* were investigated to avoid false negative results. In many studies, these patients were not excluded from the study, and this was considered a limitation in these studies.

There are similar studies evaluating the frequency of *H pylori* infection and colorectal polyps in the literature. In a study of 4466 patients conducted in Korea in 2016; *H pylori* infection was more common in patients with colorectal adenomatous polyps. In this study, the rate of *H pylori* infection was found to be higher in large adenomas and multiple adenomas. Although the rate of *H pylori* infection was found to be increased in colonic adenomas, no correlation was found between *H pylori* infection and polyp frequency in rectal polyps.^[10]^ In our study, the difference was significant in all localizations, including the rectum. In a study designed in China in 2019, 180 patients with colorectal adenoma and a total of 1195 people were examined, and the rate of *H pylori* infection was found to be higher in the group with adenoma.^[6]^ In the USA, Sonneberg et al conducted a study with 156,000 people, and observed that *H pylori* gastritis was more common in hyperplastic polyps, adenomatous polyps, advanced adenomas, high-grade dysplasia and adenocarcinomas.^[11]^

In some studies, there was no significant relationship between *H pylori* infection and colon cancer. In Turkey, Boyuk et al^[13]^ conducted a study consisting of 314 patients in which the presence of *H pylori* and the relationship between intestinal metaplasia and colon neoplasm were investigated, but no significant results could be reached. In the study of Robertson et al^[14]^ in the USA with 1794 patients, it was seen that *H pylori* infection reduced the incidence of colorectal adenoma.

The physiological mechanisms underlying the relationship between *H pylori* and colonic neoplasms are not yet fully understood. Many previous studies show that *H pylori* infection increases the serum gastrin level. Hypergastrinemia is thought to play a growth-inducing role in colonic mucosal cells.^[15–17]^ However, in some studies, no significant relationship was found between hypergastrinemia and colorectal neoplasia. It has been observed that there is no significant increase in the risk of colon adenoma and CRC development with long-term use of PPIs or in hypergastrinemia conditions such as Zolinger-Ellison syndrome.^[15]^ In the study of Robertson et al^[14]^ in the USA, no relationship was found between colorectal adenomas and gastrin levels.^[11]^ In a study designed in Germany in 377 patients aged 50 years and older, *H pylori* infection was found to be more common in patients with hyperplastic polyps and low-grade intraepithelial neoplasia. However, in this study, no significant difference was found between serum gastrin levels in *H pylori*-positive and -negative patients.^[18]^ In this study, the majority of patients with *H pylori* infection were found to have cag A virulence.^[18]^ In addition, some studies have shown that increased gastrin levels are the result of autocrine secretion of gastrin by CRC cells. It has been observed that serum gastrin levels decrease after surgical resection of the tumoral tissue. These studies suggest that the effect of gastrin on colorectal neoplasms is independent of *H pylori*.^[15,19,20]^

Some studies suggest that the microorganism is a direct activator of colorectal carcinoma.^[6,7]^ In a study by Soylu et al^[7]^ in Turkey, 21.6% of biopsy preparations of resected colon polyps were stained positive for *H pylori*. In addition, matrix metalloproteinases, whose activation is increased after *H pylori* infection, are thought to be effective in colorectal polyp development and CRC invasion.^[6,21]^ The induction of the pro-inflammatory cyclooxygenase enzyme by *H pylori* infection is also thought to contribute to the development of polyps.^[6,22,23]^ In addition, it is thought that adenomatous polyps cause an increase in T regulatory cells, and T regulatory cells cause the persistence of *H pylori* infection. According to this information, it is thought that the presence of *H pylori* and adenomatous polyps affect the development of each other.^[24,25]^

As a result of *H pylori*-associated chronic atrophic gastritis, changes in the GI microbiota may develop with decreased gastric acid secretion. Overgrowth of some microorganisms, such as B. fragilis and E. faecalis, may occur. It is thought that this situation may be effective in the development of *H pylori*-associated gastric cancer and CRC.^[15,26]^ Sobhani et al^[27]^ discovered that Bacteriodes and Provetella species were more prevalent in the gastrointestinal tracts of CRC patients.

In a study conducted in Thailand, it was reported that *H pylori* infection significantly increased the risk of developing high-grade dysplasia and CRC. In this study, it was reported that the risk of *H pylori* infection is higher in MDM2 SNP39 G/G homozygous individuals and the risk of advanced colorectal neoplasia in these patients.^[28]^

The limitation of our study is that it was retrospective, and therefore, it was not possible to reach all of the patients’ information, such as socioeconomic status, ethnicity, cigarette and alcohol consumption, physical activity, dietary habits, and nonsteroidal anti-inflammatory drugs use. The rate of *H pylori* infection was found to be 34.2% in the patients included in the study. This rate is lower than the rate of *H pylori* infection reported in Turkey. The fact that the study was a single-center study conducted in a center in the country’s capital, and thus the study was conducted in a group of patients who had access to more health services and had a better socioeconomic status, is thought to be related to this situation. The major virulence factors of *H pylori* thought to be associated with malignancy are CagA and VacA genes. In our study of *H pylori*, CagA and VagA genes were not investigated.

As a result, it has been shown that there is a significant relationship between *H pylori* and colorectal polyps. Epidemiology-based studies in larger populations will provide more detailed data on the relationship between *H pylori* and colorectal neoplasia.

## Author contributions

**Conceptualization:** Nergis Basmaci, Ali Karataş, Mustafa Ergin, Giyaseddin Şükrü Dumlu.

**Data curation:** Nergis Basmaci, Mustafa Ergin.

**Formal analysis:** Nergis Basmaci, Ali Karataş, Mustafa Ergin.

**Funding acquisition:** Nergis Basmaci, Ali Karataş, Mustafa Ergin.

**Investigation:** Nergis Basmaci, Mustafa Ergin.

**Methodology:** Nergis Basmaci, Ali Karataş, Mustafa Ergin.

**Project administration:** Nergis Basmaci, Mustafa Ergin.

**Resources:** Nergis Basmaci, Mustafa Ergin.

**Software:** Nergis Basmaci, Mustafa Ergin.

**Supervision:** Nergis Basmaci, Mustafa Ergin, Giyaseddin Şükrü Dumlu.

**Validation:** Nergis Basmaci, Ali Karataş, Mustafa Ergin.

**Visualization:** Nergis Basmaci, Ali Karataş, Mustafa Ergin, Giyaseddin Şükrü Dumlu.

**Writing – original draft:** Nergis Basmaci, Ali Karataş, Mustafa Ergin.

**Writing – review & editing:** Nergis Basmaci, Ali Karataş, Mustafa Ergin, Giyaseddin Şükrü Dumlu.
